# Clinical Outcome in Persons with Severe Mental Disorders Attending a Mental Health Day Center during the COVID-19 Pandemic

**DOI:** 10.3390/jcm13051241

**Published:** 2024-02-22

**Authors:** Angeliki Ninou, Vaios Peritogiannis, Sophia Maria Tzimogianni, Vassiliki Fotopoulou, Maria Bakola, Eleni Jelastopulu

**Affiliations:** 1 Skitali Mental Health Day Center, Society for the Promotion of Mental Health in Epirus, 44445 Ioannina, Greece; ninougr@hotmail.com (A.N.);; 2 Mobile Mental Health Unit of the Prefectures of Ioannina and Thesprotia, Society for the Promotion of Mental Health in Epirus, 44445 Ioannina, Greece; 3 Department of Public Health, School of Medicine, University of Patras, 26500 Patras, Greece

**Keywords:** mental health day center, psychiatric rehabilitation, COVID-19, severe mental illness, community psychiatry, Greece

## Abstract

Background: Studies assessing the psychological impact of the COVID-19 pandemic on populations highlight the emergence of mental health difficulties, especially if a mental health disorder is already present. Patients with severe mental illnesses (SMIs) may be even more vulnerable to the psychosocial effects of the pandemic. However, little is known regarding the possible impact of the pandemic on SMI patients supported by community-based mental health day centers. Methods: A two-year prospective study comprising 29 individuals with SMI was conducted by the Skitali Mental Health Day Center in Ioannina, Northwest Greece. The described group of examined patients consisted mainly of psychotic patients (65.5%). Patients were assessed using the Health of Nations Outcome Scale and the Global Assessment of Functioning scale, and scores prior to and after the onset of the pandemic were compared. Results: The results indicated that participants did not present any significant decline in their overall clinical status during the COVID-19 pandemic and the national lockdown measures. Conclusions: This finding is relevant because previous research has shown that the pandemic may negatively impact adherence to treatment and service attendance and that the symptomatology of patients with SMIs may further deteriorate. It is suggested that the operation of mental health day centers during collective stressful events should be preserved, but further research is needed to evaluate their role in maintaining continuity of care during such events.

## 1. Introduction

Since the declaration of COVID-19 as a pandemic by the World Health Organization (WHO) in March 2020, populations have witnessed an almost radical change in everyday functioning on a global scale. Social distancing, isolation, limited socioeconomic activity, general uncertainty about the future and prolonged disease-related fear, along with excessive information with terrorizing content, constituted what seemed to be a unique stressful event [1]. Previous research has already underlined the significant correlation between stressful life events, such as changes in social relations, job insecurity, loneliness and health concerns and psychological problems, such as anxiety, depression and distress in the general population [2]. Indeed, the existing literature suggests that restrictive measures, such as quarantine and social distancing, may have a substantial impact on the psychological well-being and emotive reactions of the population. Psychological reactions to pandemics include maladaptive or avoidance behaviors, emotional distress, anxiety, fear, frustration, anger and depression [3]. Therefore, the effects of the pandemic on the mental health of the population were rapidly pointed out by a growing body of research. In the early stages of the pandemic, a study carried out in Wuhan, China, showed that more than one-fourth of the city’s population reported increased levels of anxiety and depression, and one-third reported sleep disorders [4]. Other studies identified similar psychological difficulties [5,6], along with post-traumatic stress disorder (PTSD) symptoms [7], COVID-19-related traumatic distress [8] and high levels of COVID-19-related fear [9]. It should be noted that, although most studies have been conducted in Western, high-income countries, there is emerging evidence that in low- and middle-income countries there may also be an increase in symptoms of mental health distress and a consequent increase in the prevalence of clinically significant mental health problems [10]. Such responses to the COVID-19 pandemic may be even more relevant in those countries due to the well-documented socioeconomic and health inequities they face [10]. Moreover, a study that explored mental health manifestations in people who suffered a COVID-19 infection at one year suggested that they had an increased risk of incident mental disorders, such as anxiety disorders, depression, stress and adjustment disorders, substance use disorders, neurocognitive decline and sleep disorders compared with unaffected controls [11]. It should be noted, however, that wide variability in the prevalence of mental health problems and psychosocial consequences due to the pandemic has been observed across countries [12].

Considering the above and the limited accessibility to mental healthcare imposed by the circumstances [13], it was rather expected that individuals with a pre-existing mental health disorder, especially a severe one, would suffer a greater impact. Given the cognitive, psychological and socio-economic challenges that people with mental health conditions usually face, fears were expressed about possible exacerbation of pre-existing conditions [14,15,16,17,18,19]. Evidence from around the world seems to support the aforementioned concerns. In two studies at the peak of the pandemic in Spain, participants presenting a pre-existing mental health disorder reported higher levels of anxiety [20] and depression [21] than healthy controls; similar results were obtained in Serbia [22]. At the peak of the first wave of the COVID-19 pandemic in China, with strict lockdown measures, patients with previous psychiatric diagnoses (mostly depressive and anxiety disorders) had significantly higher levels of anxiety, depression and stress compared to the general population, and more than one-quarter of them reported PTSD-like symptoms and insomnia [23]. A study in Canada involving a large sample of participants found that the rates of depressive symptoms and symptoms of generalized anxiety disorder were high. Importantly, the proportion of participants with clinically significant worsening in anxiety, depression and suicidal ideation was higher in those with pre-existing psychiatric problems than in those without a psychiatric history [24]. These findings were replicated in a subsequent, very large study with 55,589 participants from 40 countries, in which probable depression was detected in 17.8% of the sample and distress was detected in 16.7%. Notably, persons with a history of mental disorders had higher rates of current depression (31.8% vs. 13%) [25].

With regard to patients with severe mental illness (SMI), there were early concerns regarding the impact of the pandemic on those patients’ physical and mental health, as well as concerns about the continuity of care in this patient population [16]. It has been shown that patients with SMI have higher rates of physical morbidity and premature mortality due to natural and unnatural causes than the general population [26,27]. Moreover, they are more likely to suffer a COVID-19 infection and display significantly higher mortality rates than the general population [28,29]. However, less is known about the impact of the pandemic on the mental health of patients with SMIs. In a study in India, 29.5% of the participants presenting with a severe mental health disorder reported an exacerbation of symptoms during the early stages of the pandemic, and between 20% and 37.9% of them reported problems with sleep, food intake and personal care [30]. Similarly, symptom exacerbation, along with anxiety and fear, was reported by a study on psychiatric outpatients (>40% with SMI) in Italy and Paraguay [31]. In the USA, in a study carried out from July to October 2020, participants with schizophrenia or schizoaffective disorder reported an exacerbation of negative symptoms, such as anhedonia, avolition and asociality [32]. In England, a study of 34,446 patients with severe mental health disorders reported an overall deterioration in their mental health state during the first national lockdown [33]. Finally, a very recent review of the evidence suggested that the pandemic affected many aspects of schizophrenia patients’ symptomatology, mostly negative symptoms, but also adherence to treatment and service attendance [34]. It has been further suggested that patients with schizophrenia may be vulnerable to the effects of the COVID-19 pandemic due to several risk factors, such as social problems and disease-related factors [35].

Accordingly, a survey in 130 countries by the WHO suggested that the demand for mental health care had increased over the COVID-19 pandemic but also that the pandemic had negatively impacted access to mental health services. At the same time, mental health services had been disrupted in 93% of countries worldwide [36]. Patients’ treatment attendance and engagement with services had also been affected by the pandemic and had decreased, while patients were less likely to receive timely outpatient care because community and rehabilitation services had been considerably reduced or had even ceased [37].

The psychological impact of the COVID-19 pandemic on the Greek population have been highlighted by several studies involving people with common mental disorders. Being on psychiatric medication was associated with higher COVID-19-related fear [9], while a previous history of depression was associated with the development of depressive symptomatology [38]. Finally, post-traumatic stress symptoms were linked to previous psychiatric histories [39]. Less is known regarding the psychosocial impact of the pandemic on patients with SMI in Greece and the interventions that could ameliorate its negative effects on this vulnerable population. The present study is therefore relevant in that it aims to explore the psychological impact of the pandemic on patients with SMI continuing to receive support from a community-based psychosocial rehabilitation service in Greece during the pandemic. To the best of our knowledge, very few studies have specifically examined the experiences of people receiving community-based mental healthcare services during the pandemic. The question seems important, considering that it adds to the as-yet limited research on the psychological impact of a collective stressful event on a particularly vulnerable population; it also contributes to the discussion about the role of community-based mental health day centers during psychosocial stressful events. Given the organizational flexibility and personalization of the interventions delivered by community-based mental health services, it could be hypothesized that they could play a major role in an event such as the pandemic [40]. Thus, another objective of the present study was to better understand how to optimize support for people with chronic and severe mental disorders during a collective stressful event.

## 2. Materials and Methods

### 2.1. The Study Setting

The Skitali Mental Health Day Center (Skitali MHDC) in Ioannina, Northwest Greece, is a community-based psychosocial rehabilitation service providing free daily individual counseling, home-based interventions and group rehabilitation activities to people with severe mental disorders. Addressing its services to approximately 120,000 inhabitants in its area, the center accepts individuals referred by psychiatrists in both public and private practices. The center’s population presents a variety of psychiatric diagnoses along with significant psychosocial difficulties, with schizophrenia and related psychotic disorders being the most prevalent. People with substance abuse disorders and moderate to severe mental retardation are not treated by the center, but are referred to other services in the area.

### 2.2. Study Design

A prospective study was conducted in Skitali MHDC in Ioannina, Greece. As our study aimed to determine possible psychological impacts of the pandemic on the center’s population, we opted for data obtained before and after national lockdown measures issued in March 2020. Data from the end of the second semester of 2019 represented the baseline information, while data from the end of the second semester of 2020 and from that of 2021 served, respectively, as 12-month and 24-month follow-up evaluations. All patients who attended the Skitali MHDC at the end of 2019 were initially eligible for participation in the study. Patients were included in the study if they regularly attended the day center over the two-year study period. Patients who disengaged from the service and those who attended the day center only intermittently over the two-year follow-up were excluded from the analysis, since there were missing measurements of their symptomatology and functioning. Patients who first referred to the center after the onset of the pandemic were also excluded, since they had no baseline assessment.

The study was approved by the Institutional Board following the Institution’s research guidelines. The need for patients’ informed consent was waived since the study was not interventional and the measurements were regular parts of patients’ routine assessments.

### 2.3. Participants and Measurements

Participants in the study included all individuals with a psychiatric diagnosis according to the International Classification of Diseases, 10th Revision (ICD-10), attending the center’s program from the second semester of 2019. During the second semester of 2019, a mean monthly number of 50 individuals attended the day center’s program. Due to lockdown measures, continuity of physical attendance was not possible, and routine treatment plans were halted or significantly altered. Changes in nature and means of support were required, comprising daily distance communication and virtual group activities, as well as in-person individual consultations when considered necessary. Following the progressive relaxation of lockdown restrictions between June 2020 and November 2020, support also included in-person participation in small groups, taking place in outdoor spaces. Given the different phases of the pandemic and the constant changes in services offered, only 29 individuals who were supported continuously until the end of the second semester of 2021 were included in this study.

Data were collected from mental health assessments carried out in our center every six months, such as the Health of the Nation Outcome Scales (HoNOS) and the Global Assessment of Functioning scale (GAF). HoNOS is a 12-item instrument measuring the clinical symptoms and social functioning of people aged 18–64 years. Each item is rated on a five-point scale, with higher points indicating an increased level of impairment. GAF measures the severity of clinical and social functioning impairment on a scale ranging from 0 to 100, with a lower score indicating a more severe impairment.

Both assessment measures are routinely used by the center’s psychologists following the institution’s quality of care guidelines. Thus, instrument selection was based on measures already available in Skitali MHDC, opting for data obtained before the onset of the pandemic. The HoNOS presents good validity and, despite its variable reliability, is considered better than other measures for mental health settings, covering a wide range of areas of overall functioning [41]. The HoNOS has commonly been used to assess clinical and functional changes in patient populations over time [42]. In the United Kingdom, the use of the HoNOS is recommended for regular use in clinical practice by the authorities [43], while in some regions its collection is mandatory; accordingly, the HoNOS is widely used in service evaluation research [42]. Most importantly, it has been used in research on patients with schizophrenia-spectrum disorders being treated in community mental healthcare services [44]. The validity and reliability of the Greek version of the HoNOS [45] was tested in the Greek population and was reported to be high [46]. The GAF scale shows poor inter-rater reliability and poor discriminant validity, especially in the outpatient population [47]. However, we chose to include the GAF results in this study, as the instrument is widely used in mental health settings and may provide additional information for our research, at least at a clinical level. Moreover, it has been shown that GAF ratings can be used to measure changes and outcomes at the group level, which is relevant to the present study [48]. The GAF scale has proven validity and reliability in the Greek language [49]. Both scales have been previously used in research in community mental healthcare settings in Greece [50,51].

### 2.4. Statistical Analysis

Data were analyzed using the Statistical Package for the Social Sciences (SPSS) version 23.0 (SPSS Inc., Chicago, IL, USA) for Windows. Descriptive statistics were used to summarize all variables. Analysis of HoNOS results concerned the scores of both individual items and subscales due to concerns about the validity of sum scores and dimensionality of the subscales [42,52,53].

Due to the small sample size, normality tests were performed for HoNOS and GAF scores using the Shapiro–Wilk test and inspecting the level of skewness and kurtosis in data distribution. Only the total HoNOS score for 2019 and the HoNOS Social subscale and GAF scores for 2020 were normally distributed. All other data were highly skewed and the Shapiro–Wilk test presented *p* values below 0.05, indicating a non-normal distribution.

Due to the small sample size and the non-normal distributions, we decided to run a non-parametric repeated measures test, the Friedman test, in order to estimate differences in HoNOS and GAF scores over three time points.

## 3. Results

Twenty-nine individuals attending the day center’s program were enrolled according to the inclusion criteria of this study. The participants’ mean age was 43.7 years and most were men (58.6%). Most of the participants were allocated a disability pension (51.7%), a small proportion had a paid job (13.8%) and one-fifth were unemployed. The majority of the participants had a postsecondary education (86.1%). Almost half were living with their parents or siblings (48.3%) and less than one-fifth were living alone. One-fourth of the patients were residents in rehabilitation facilities. Most had been attending the day centers’ program before the second semester of 2019 (79.3%). Diagnoses of schizophrenia, schizotypal or delusional disorder were prevalent (65.5%) (Table 1).

The mean scores of HoNOS and GAF results in the second semester of 2019 were 10.4 and 64.3, respectively. The HoNOS Behavior subscale presented the lowest severity for the majority of the participants (89.6%), whereas the Social subscale presented moderate to severe problems for most of them (82.5%). The HoNOS Symptom subscale indicated a need for intervention in most of the cases (72.2%) for problems ranging from mild to severe (Table 2).

Comparing the HoNOS and GAF scores obtained before, during and after the pandemic using a Friedman test, no significant difference was found (Table 3).

## 4. Discussion

In the present study, we examined whether individuals attending a community-based mental health day center presented a decline in their clinical status during the COVID-19 pandemic and the national lockdown measures. The analysis shows that those who continued to receive support from the Skitali MHDC presented no significant overall symptom exacerbation or decline in their mental health status during the two years of the pandemic. In fact, none of the HoNOS scale items and none of the GAF scores changed significantly, suggesting that patients attending the Skitali MHDC remained rather stable in terms of psychopathology and daily functioning during the period examined.

At the clinical rather than the statistical level, according to the GAF mean ranks, participants showed a slight amelioration in their overall clinical status in 2020, which declined in 2021, almost reaching the pre-pandemic levels. Again, it seems that the participants maintained a rather stable overall condition. HoNOS cognitive impairment mean rank scores seem to represent an amelioration in this domain, despite what would be expected given the circumstances. Agitated behavior, on the other hand, presented a slight increase in 2020 and remained increased in 2021. The only score presenting a stable decline throughout the two-year period was that of the behavior subscale, indicating that the participants experienced increased behavior problems. However, as we have already pointed out, there are methodological concerns with the use of the HoNOS subscales due to suspected multidimensionality.

The main findings of the present study, however, do not agree with the general literature concerning the psychological impact of the pandemic on psychiatric populations, although we should consider that most of the studies reporting exacerbation of symptoms were cross-sectional [54] and concerned the first wave or the first year of the pandemic [13]. From that aspect, our finding concerning the increase of agitated behavior during the first year of the pandemic agrees with similar findings during the same time period [33]. Nevertheless, a small portion of the studies found no significant psychological impact on psychiatric patients, even during the first stages of the pandemic. Comparing pre- and post-pandemic data, two studies [54,55] not only reported a lack of significant symptom exacerbation in patients with psychotic disorders but also an amelioration in some aspects of their daily functioning. These studies seem to corroborate our findings, especially concerning the amelioration in GAF scores during 2020 and the constant amelioration of cognitive impairment.

According to the aforementioned studies, resilience factors, such as practicing religiosity, receiving formal updates about the pandemic and having the ability to draw upon pre-pandemic social networks during the pandemic, could explain their results. In the present study, participants were systematically informed about the pandemic based on official reports and guidelines of protection, especially through virtual group meetings. This could have enhanced some sense of control and maybe procured a sense of meaningfulness in this out-of-the-ordinary situation. As has already been suggested, people with mental health disorders could be significantly impacted by stressful events that they perceive as being out of their control [56].

In addition, our data concern individuals who attend, usually for years, a community-based psychosocial rehabilitation service. Attendance at such a service provides the means for building a social support network through group activities and socializing, as well as access to at-home interventions. During the lockdown measures, support continued to be provided in our center through individual or group activities at a distance, and patients regularly communicated with each other as well as with the therapists through phone and video calls. This permitted the maintenance of social support, even in an altered form. Evidence from previous research has already underlined the importance of social networks and support to recovery [57,58,59]. The authors of a study in Portugal, carried out from March to May 2021, reached the same conclusions: Participants mostly diagnosed with schizophrenia and other psychotic disorders attending psychosocial rehabilitation services and thus continuing to draw upon their rehabilitation communities during the pandemic were found to report medium to high levels of resilience and mental well-being [60].

It could be argued that modalities of social support offered by such services during the pandemic are not of the same quality as pre-pandemic realities, given the lockdown restrictions and, in many instances, the lack of at-distance means to ascertain accessibility [37]. However, interviews with patients having a schizophrenia diagnosis suggest that, despite the possible initial psychological impact of the situation, maintaining continuity of care and even a limited form of contact could help patients better adjust [61].

Another point to consider is the willingness of patients with SMI to use all available resources to protect their health and preserve their well-being during the pandemic, which could explain their attendance at day centers. A recent qualitative study addressed the experiences of coping with COVID-19 among patients with schizophrenia in Korea and found that routine care was interrupted on several occasions, while patients experienced a dual threat from their mental illness and the emerging infectious disease, along with inequity and paradoxical situations. Still, they were able to employ various strategies and self-help measures to cope with pandemic stress, and importantly, kept seeking support from mental health professionals [62]. Another recent study in Turkey concerning patients with schizophrenia found that the use of available resources, adaptation to changes in daily living, and social interaction and support were facilitators in coping with the pandemic burden [63]. These observations are indirectly supported by a recent review regarding the concerns and fears of patients with mental disorders toward COVID-19 vaccination, which highlights that, contrary to expectations at the beginning of the pandemic, SMI may positively affect attitudes toward the vaccine [64]. This finding suggests that the ability to seek support, which may influence adherence, could represent a crucial protective personality trait in these patients. Notably, patients with SMI in our catchment area displayed high rates of COVID-19 vaccination [65].

Possible confounding factors that could have affected the observed outcomes in patients should be considered. For instance, if patients received further support from services during the pandemic, this might have partly accounted for the stabilization of their clinical state. However, it is known that, globally, the response of the mental health systems to the pandemic included the reduction of outpatient appointments, the treatment of emergencies only and the minimization of caseloads [66]; this was also the case in our area. Thus, it seems highly unlikely that the present sample of patients received any additional pandemic support during the study period, and their attendance at the Skitali MHDC was probably their best opportunity to continue receiving mental health care.

### 4.1. Limitations and Strengths

The main strength of the present study is its prospective design and relatively long follow-up, addressing the participants’ symptomatology and functioning over different phases of the COVID-19 pandemic. However, the exploration of psychological responses to the pandemic, such as fear and worry, as well as the assessment of patients’ well-being, was beyond the scope of the study. Indeed, some studies on psychiatric patients, including those receiving community-based support, recorded increased distress, loneliness, fear and anxiety, and perceived risk to mental health and well-being, even in the absence of any exacerbation of psychiatric symptoms [67,68,69,70]. Whether these psychological effects would contribute to an eventual deterioration of patients’ mental health in the long term remains to be studied [69]. It remains an important aspect of the pandemic that needs to be taken into account, considering that the world, two years after its onset, still faces constant resurgences and relative fears.

Several other limitations are present in our study. First, it concerns a small sample of patients, who may not represent the whole patient population, and only one community-based rehabilitation service. This restricted the statistical processing of the data, and the results lack generalizability, as they may not describe the actual impact of the pandemic on people who regularly attend similar services. In addition, even though considerable effort was made to reach every person normally using our service and offer the means to receive further support (assistance in technological means use, lending relative material, etc.), some people, especially those with severe physical comorbidities and those living in distant rural areas, could not or would not access our services beyond phone-based communication, and they eventually dropped out of the program during the first year of the pandemic. This could mean that we could not assess the psychological state of people who were possibly presenting higher levels of psychosocial need than the study’s participants. Finally, the lack of a control group of patients who did not attend a community-based psychiatric rehabilitation service during the pandemic does not allow us to ascertain the role of the Skitali MHDC in the stabilization of the clinical and functional state of the participants.

### 4.2. Potential Implications and Future Research

The findings of the present study may be relevant to clinical practice and psychiatric rehabilitation and may have potential policy implications for mental health services in health or other crises. It has been argued that the social distancing imposed by the pandemic can cause significant emotional distress to patients with SMIs, possibly leading to a relapse of psychotic symptoms in this population and thus to an increased risk of rehospitalization [71]. It has also been shown that during the pandemic, the rate of involuntary admissions increased significantly due to patients’ non-adherence to treatment and disengagement from mental health services, both further associated with the exacerbation of symptoms and relapse [72,73,74]. It appears that a day center can effectively retain patients with SMI in a community-based, broad psychosocial rehabilitation program, even modified to conform to the pandemic, thus preventing the deterioration of their mental health and subsequent relapses and hospitalizations. Accordingly, the operation of day centers and other community-based mental health services should be preserved over periods of stressful events as a means of maintaining continuity of care in this vulnerable population.

The present study includes patients with severe and chronic mental disorders who regularly attend a community-based rehabilitation service. Those patients were eligible for the day center due to their psychosocial difficulties and modest prognosis that required rehabilitation. It is not known what the impact of the pandemic may be on remitted patients, who may have had active social and vocational lives prior to the pandemic. Such patients may not wish to be followed up [75], may not use rehabilitation services and may not participate in research. Similarly, patients with poor prognosis who do not attend treatment or rehabilitation services may have been affected by the pandemic as well, but the extent of their difficulties cannot be easily assessed. These important issues need to be addressed in future research. Since the long-term pandemic effects on patients with mental disorders are currently largely unknown, further research on this topic is warranted. The role of mental health day centers caring for patients with SMIs during other collective stressful events should also be studied.

## 5. Conclusions

The present study showed that individuals with chronic and severe mental disorders attending a community-based mental health day center and receiving psychosocial treatment and rehabilitation services during the two years of the COVID-19 pandemic presented no significant decline in their overall mental health status. This led us to suggest that a day center may play an important role in promoting patients’ psychosocial functioning despite general conditions. Further research is needed to assess the role of a community-based mental health day center during collective stressful events.

## Figures and Tables

**Table 1:** Participants’ demographic and clinical characteristics.

Variables	N (%) or Mean ± SD
Total number of participants	29
Age (years) (mean ± SD)	43.72 ± 11.53
**Gender (N, %)**	
Male	17 (58.6%)
Female	12 (41.4%)
**Education (N, %)**	
Secondary School	4 (13.8%)
Vocational School	5 (17.2%)
High School/College	11 (37.9%)
University	9 (31.0%)
**Employment (N, %)**	
Employed	4 (13.8%)
Not employed	6 (20.7%)
Disability pension	15 (51.7%)
Retired	4 (13.8%)
**Cohabitation status (N, %)**	
Living alone	5 (17.2%)
Parents/siblings	14 (48.3%)
Partner/children	2 (6.9%)
Roommates	8 (27.6%)
**ICD-10 Diagnosis (N, %)**	
F20–F29 (Schizophrenia, schizotypal and delusional disorders)	19 (65.5%)
F30–F39 (Affective disorders)	2 (6.9%)
F80–F89 (Pervasive and specific developmental disorders)	2 (6.9%)
Multiple diagnosis	4 (13.8%)
Other	2 (6.9%)
**Housing (N, %)**	
Community	22 (75.9%)
Psychiatric Rehabilitation Residence	7 (24.1%)
**Attendance before second semester 2019**	
Yes	23 (79.3%)
No	6 (20.7%)

**Table 2:** HoNOS results expressed in percentages in the second semester of 2019.

HoNOS Items	Minor to no Problems	Mild Problems	Moderate to Severe Problems
Aggression/overactivity	89.6	10.4	-
Self-harm	100.0	-	-
Substance abuse	96.6	3.4	-
Cognitive impairment	62.0	20.7	17.3
Physical impairment	82.8	10.4	6.8
Hallucinations/delusions	75.9	17.2	6.9
Depressed mood	93.2	3.4	3.4
Relationships	34.5	44.8	20.7
Daily living	31.0	48.3	20.7
Living conditions	82.1	10.3	6.8
Occupation/activities	31.0	48.3	20.7
**HoNOS subscales**			
Behavior	89.6	7.0	3.4
Impairment	55.3	20.7	24.0
Symptom	27.6	17.2	55.2
Social	6.9	10.3	82.8
**Other mental/behavioral problems**	**Prevalence of problems**		
Obsessive/compulsive	24.1		
Anxiety	17.2		
Mental strain/tension	13.8		
Phobic	3.4		
Sleep	6.9		
None	34.5		

**Table 3:** Differences in HoNOS and GAF scores before, during and after the pandemic and their significance level according to Friedman Test.

HoNOS Items	Mean Rank			Friedman Test	
	2019	2020	2021	X^2^	*p*
Aggression/overactivity	1.93	2.03	2.03	1.043	0.658
Self-harm	-	-	-		
Substance abuse	1.97	1.95	2.09	2.923	0.407
Cognitive impairment	2.07	1.97	1.97	0.571	0.790
Physical impairment	1.97	1.98	2.05	0.636	0.818
Hallucinations/delusions	1.91	1.95	2.14	2.450	0.360
Depressed mood	2.10	2.00	1.90	3.000	0.277
Other mental/behavioral problems	1.93	2.09	1.98	0.677	0.727
Relationships	2.17	1.90	1.93	3.234	0.204
Daily living	2.10	1.88	2.02	1.344	0.526
Living conditions	1.95	1.97	2.09	1.652	0.503
Occupation/activities	1.97	1.93	2.10	1.333	0.542
**HoNOS subscales**					
Behavior	1.90	1.98	2.12	2.688	
Impairment	1.98	2.02	2.00	0.040	
Symptom	1.97	2.03	2.00	0.107	
Social	2.09	1.76	2.16	3.595	
**GAF score**	1.77	2.21	2.02	3.413	0.181

## Data Availability

Data are kept in the patients’ electronic charts of the “Skitali” Mental Health Day Center and are confidential.
